# Unmasking rare thalassemia variants through whole-exome sequencing in Huadu District, China: An observational study

**DOI:** 10.1097/MD.0000000000049002

**Published:** 2026-05-29

**Authors:** Run Guowei, Jiang Yan, Xu Jingxia, Jiang Changlv, Zeng Lihua, Yu Bizhen, Bi Jingnan, Tan Cuijin, Huang Yulan, Lei HaoHao, Ji Linhua

**Affiliations:** a Department of Hematology, Huadu District People’s Hospital of Guangzhou, Guangzhou, China; b Huadu Institute of Medicine, Guangzhou, China; c Clinical Research Center, Xuan Wu Hospital, Capital Medical University, Beijing, China; d Department of Hematologic Oncology, State Key Laboratory of Oncology in South China, Collaborative Innovation Center for Cancer Medicine, Sun Yat-sen University Cancer Center, Guangzhou, China; e Department of Laboratory Medicine, Huadu Institute of Medicine, Guangzhou, China; f Huadu District People’s Hospital of Guangzhou, Guangzhou, China; g Third Clinical School of Medicine, Southern Medical University, Guangzhou, China.

**Keywords:** genetic screening, Guangzhou, *HBB*, INDELs, SNVs, thalassemia, whole-exome sequencing

## Abstract

In regions with a high prevalence of thalassemia, conventional diagnostic methods may fail to detect atypical or complex genetic variants. Whole-exome sequencing (WES) provides a comprehensive strategy to identify such variants, allowing more accurate genotype–phenotype correlation. Nevertheless, its optimal integration into clinical workflows and its incremental value over standard diagnostic approaches remain uncertain. This study aimed to evaluate the clinical utility of WES in a heterogeneous patient cohort and to propose a framework for its rational use in endemic settings. WES was performed on 21 patients with clinically suspected thalassemia from Huadu District, Guangzhou, stratified by severity. Variant analysis encompassed both coding and noncoding regions. On average, 5440.7 ± 94.3 insertions/deletions and 49,719.3 ± 492.5 single-nucleotide variants per individual were detected. Frameshift insertions/deletions were predominantly localized to the *HBB* gene. Noncoding single-nucleotide variants in 3′ untranslated regions were associated with reduced mean corpuscular hemoglobin (HGB) concentration (301 ± 25 g/L in patients with vs 321 ± 18 g/L in those without 3′ untranslated region variants, *P* = .008). Compound heterozygosity involving classical β-thalassemia mutations (CD41/42(-TTCT) β^0^ and IVS-II-654(C>T) β^+^) accounted for 85.7% of severe cases. Three rare non-β-globin variants were identified: *MYB* c.107G>A (p.R36H) in 1 case, a variant of uncertain significance potentially linked to fetal HGB regulation; *HBD* c.440A>T (p.H147L) in another, which produced an artificially normalized HbA_2_ value (3.1%), creating a risk of β-thalassemia misdiagnosis; and *HBG1* c.364G>T (p.E122*) in a third case, co-occurring with α-thalassemia and iron deficiency. HGB levels declined significantly with increasing clinical severity (mild: 99.7 ± 2.5 g/L; very severe: 31.0 ± 26.1 g/L, *P* < .05). WES proved to be a powerful diagnostic tool, identifying causative or contributing genetic variants in all patients in this cohort, including those with complex or atypical presentations. Although it may not yet be feasible as a first-tier screening approach due to cost constraints, our findings strongly support the selective integration of WES into existing thalassemia diagnostic workflows in endemic regions. Such an approach enhances diagnostic precision, clarifies complex genotypes, and can guide more individualized management strategies.

## 1. Introduction

Thalassemia poses a significant public health challenge in Guangdong Province, China. Provincial carrier rates exceed 6.8%, with some studies estimating an overall frequency approaching 11%. Significant disparities exist; Huadu District reports a prevalence of approximately 8.3%.^[1]^ In contrast, Dongguan shows α-thalassemia and β-thalassemia carrier rates of 7.6% and 3.8%, respectively.^[2]^ In contrast, Western regions such as Yangjiang exhibit exceptionally high overall frequencies, reaching up to 20%.^[1]^ These figures are further compounded by population migration and genetic heterogeneity.

Conventional diagnostic techniques, including hemoglobin (HGB) electrophoresis, gap polymerase chain reaction, and multiplex ligation-dependent probe amplification, are limited to detecting predefined common mutations. Province-wide studies have estimated that 10% to 20% of thalassemia carriers remain undiagnosed using conventional methods due to rare variants.^[1,2]^ These methods often fail to detect noncoding variants, novel mutations, structural rearrangements, or variants beyond their target regions (deep intronic or regulatory single-nucleotide polymorphisms [SNPs]),^[1–3]^ leading to diagnostic omissions, particularly among silent carriers and those with atypical mutations.

Whole-exome sequencing (WES) provides a comprehensive alternative, enabling investigation of the entire protein-coding genome. This facilitates the detection of a broad spectrum of pathogenic variants, including coding, splice-site, insertions/deletions (INDELs), and regulatory single-nucleotide variants (SNVs), in both globin (*HBA1/2*, *HBB*) and modifier genes (*BCL11A, KLF1*).^[4]^ Consequently, WES enhances diagnostic accuracy by identifying variants undetectable by traditional methods, thereby providing a stronger foundation for genetic counseling and prenatal diagnosis.^[1]^

Despite extensive provincial screening, WES has not been implemented in high-risk subregions such as Huadu District, where carrier rates reach 8.3%. This study represents the first comprehensive WES-based profiling of rare thalassemia variants in Huadu District, decoding a unique mutational spectrum distinct from provincial patterns.

To address this gap, we conducted a study to evaluate the diagnostic yield of WES in a consecutive cohort of patients with a high clinical suspicion of thalassemia in Huadu District. Unlike prior studies that limited WES to cases negative on conventional testing, we applied WES broadly to delineate the comprehensive genetic landscape, identify the proportion and types of variants missed by standard diagnostic methods, and critically evaluate the clinical scenarios in which WES offers the greatest incremental value. Our findings establish a model for precision prevention in endemic regions and provide a framework for cost-effective WES integration in resource-limited settings.

## 2. Patients and methods

### 2.1. Patients

This retrospective observational study included 21 patients with a high clinical suspicion of thalassemia, recruited from Guangzhou Huadu District People’s Hospital. To comprehensively evaluate the underlying genetic landscape and directly compare the diagnostic capability of WES against the current standard of care in a real-world clinical setting, we applied WES to a consecutive series of clinically suspected cases irrespective of their prior conventional testing results.

Inclusion criteria were clinical suspicion of thalassemia based on hematological indices (microcytic, hypochromic anemia with reduced mean corpuscular volume (MCV) and mean corpuscular HGB [MCH]) or otherwise unexplained anemia; residence in Huadu District. Exclusion criteria were recent blood transfusion (<3 months), the presence of other diagnosed hematological disorders, and unwillingness to provide informed consent.

This consecutive-case design allowed us to assess the full potential of WES, including its ability to confirm and characterize cases already positive by conventional methods and to resolve cases in which conventional results were negative or equivocal. The study was conducted in accordance with the Declaration of Helsinki. All participants or their legal guardians provided written informed consent before inclusion. Ethical approval was obtained from the Institutional Review Board of the Huadu District People’s Hospital of Guangzhou (Approval No. 2021117).

The cohort comprised 11 females and 10 males, aged 14 to 73 years. Patients were stratified into 4 groups based on disease severity as determined by HGB levels:

Mild (M): HGB > 90 g/L (n = 3);
Moderate (Mo): HGB 60 to 90 g/L (n = 7);
Severe (S): HGB 30 to 60 g/L (n = 8);
Very severe (VS): HGB < 30 g/L (n = 3).

Iron status was assessed using serum ferritin and iron levels in all patients to account for the potential confounding effects of iron deficiency. All patients underwent comprehensive laboratory evaluation, including a complete blood count and iron studies. Demographic and clinical characteristics are summarized in Table 1.

**Table 1:** Basic patient information.

No.	Gender	Age	Graded	HGB (g/L)	HCT (%)	MCV (fL)	MCH (pg)	MCHC (g/L)	Ferritin (ng/mL)
FXY	Female	46	M	100	31.2	62.8	20.1	321	247.2
LZR	Male	73	M	99	30.6	69.4	22.4	324	371.1
YJW	Male	38	M	104	34.6	50.4	15.1	301	231.3
ZHS	Female	26	Mo	88	29.2	63.3	19.1	301	7.7
ZZF	Male	25	Mo	79	23	83	27.5	343	341.2
BWS	Male	14	Mo	98	30	89.7	29.2	326	1650
QZY	Female	14	Mo	99	31.3	88.4	28	317	1650
WKT	Male	20	Mo	84	27.8	72	21.8	302	1034
YGX	Male	55	Mo	77	22.5	56.8	19.4	342	2804.6
PCH	Female	31	Mo	81	28.6	52.8	15	285	1.9
CXM	Male	22	S	39	13.7	72.5	20.6	285	280.4
DDN	Female	27	S	51	16.9	73.2	22.1	302	341.2
FDQ	Female	63	S	53	22.5	79	20.3	236	723.3
ZWZ	Female	43	S	60	19.2	72.7	22.7	313	1018
SY	Male	18	S	56	16.7	70.5	23.6	335	1403.8
LRX	Female	13	S	56	16.5	72	24.3	337	301
CWY	Female	19	S	49	14.8	74.3	24.7	332	1562.7
HWL	Female	14	S	55	19.8	76.7	21.3	278	3790.5
HDC	Male	29	VS	21	6.2	51.2	17.4	339	1293
LJC	Male	14	VS	9	3.2	64	18	281	220.8
CHH	Male	19	VS	63	20.7	79.8	26.5	304	284.6

HCT = hematocrit, HGB = hemoglobin, M = mild, MCH = mean corpuscular hemoglobin, MCHC = mean corpuscular hemoglobin concentration, MCV = mean corpuscular volume, Mo = moderate, S = severe, VS = very severe.

### 2.2. Blood count analysis

Peripheral blood samples (2 mL) were collected from each participant into ethylenediaminetetraacetic acid-anticoagulated tubes. Complete blood count analyses were performed using the Sysmex XN-1000 automated hematology analyzer (Sysmex Corporation). The measured parameters included MCV, MCH, mean corpuscular HGB concentration (MCHC), and HGB concentration.

### 2.3. WES, data processing, and analysis

Peripheral blood samples (2 mL) were transported to the DAAN Clinical Laboratory Center (Guangzhou, China) for processing. WES was performed on the Illumina NovaSeq 6000 platform (Illumina), generating 150-bp paired-end reads with an average coverage depth of 100×. Variant filtering and prioritization were conducted as follows: retention of variants with a minor allele frequency <0.1% in the Genome Aggregation Database East Asian population; prioritization of coding, splice-site, and regulatory variants in globin-related and modifier genes, including *HBA1/2*, *HBB*, *HBD*, *HBG1*, *MYB*, *BCL11A*, and *KLF1*; prediction of functional impact using in silico tools (sorting intolerant from tolerant, PolyPhen-2, and combined annotation-dependent depletion, with a threshold >20); cross-referencing with established thalassemia databases and ClinVar; and manual review of candidate variants in the context of clinical phenotypes. The pathogenicity of identified variants was classified according to the American College of Medical Genetics and Genomics guidelines.^[5]^

#### 2.3.1. Data preprocessing

Raw sequencing data generated by the Illumina platform were processed using fastp (v0.12.6) to remove adapter sequences and low-quality reads, producing high-quality clean data for downstream analyses.

#### 2.3.2. Sequence alignment and processing

Quality-filtered reads were aligned to the reference genome using Sentieon BWA with default parameters. Post-alignment processing, including read sorting and duplicate removal, was performed using the Sentieon driver. Mapping quality metrics, including sequencing depth and coverage, were calculated using bamdst.

#### 2.3.3. Variant calling and analysis

Initial variant calling for SNPs and INDELs was performed using Sentieon DNAseq. Somatic SNPs and INDELs were identified using Mutect2, whereas somatic copy number variations were detected using CNVkit. All identified variants were functionally annotated using ANNOVAR.

#### 2.3.4. Mutational signature analysis

Somatic mutation features were extracted and analyzed using the R package Sigminer, enabling characterization of mutational patterns.^[6]^

#### 2.3.5. Quality control and variant confirmation

Independent experimental validation (e.g., Sanger sequencing) was not performed for the WES-detected variants. To ensure the accuracy of variant calls, a multi-step bioinformatic quality control process was implemented. The high average coverage depth of 100× provided a robust foundation for reliable base calling. All prioritized rare and novel variants were manually reviewed using the Integrated Genomics Viewer to visually inspect sequencing read alignments and verify the presence of each variant, thereby reducing the likelihood of false positives arising from sequencing or alignment artifacts. We explicitly acknowledge the lack of orthogonal validation as a technical limitation in the Discussion.

### 2.4. Statistical analysis

Genotypic profiling results of thalassemia, including variant spectrum characterization and genotype frequency distribution, were systematically analyzed as the primary study endpoint. Continuous variables are reported as mean ± standard deviation (SD). For between-group comparisons, we performed a one-way analysis of variance, followed by Tukey’s post hoc test for multiple comparisons (IBM SPSS Statistics, version 25.0; IBM Corp.). An unpaired *t* test was used for the specific comparison of MCHC in patients with and without 3′ untranslated region (UTR) variants. A *P*-value < .05 (two-tailed) was considered statistically significant.

## 3. Results

### 3.1. Hematological characteristics by disease severity

A summary of key clinical and hematological parameters for all 21 cases, including iron study results, is provided in Table 1. This comprehensive profiling was essential for accurate genotype–phenotype interpretation. A significant inverse correlation was observed between disease severity and HGB levels (*P* < .05). HGB concentrations declined progressively across severity groups: the M group (99.67 ± 2.52 g/L) maintained near-normal values, while the Mo (86.14 ± 8.01 g/L), S (53.38 ± 5.72 g/L), and VS groups (31.00 ± 26.11 g/L) demonstrated increasingly impaired erythropoiesis. Notably, the VS group exhibited substantially greater HGB variability (SD: 26.11 g/L) compared with the other groups (SD range: 2.52–8.01 g/L).

**Figure 1.**
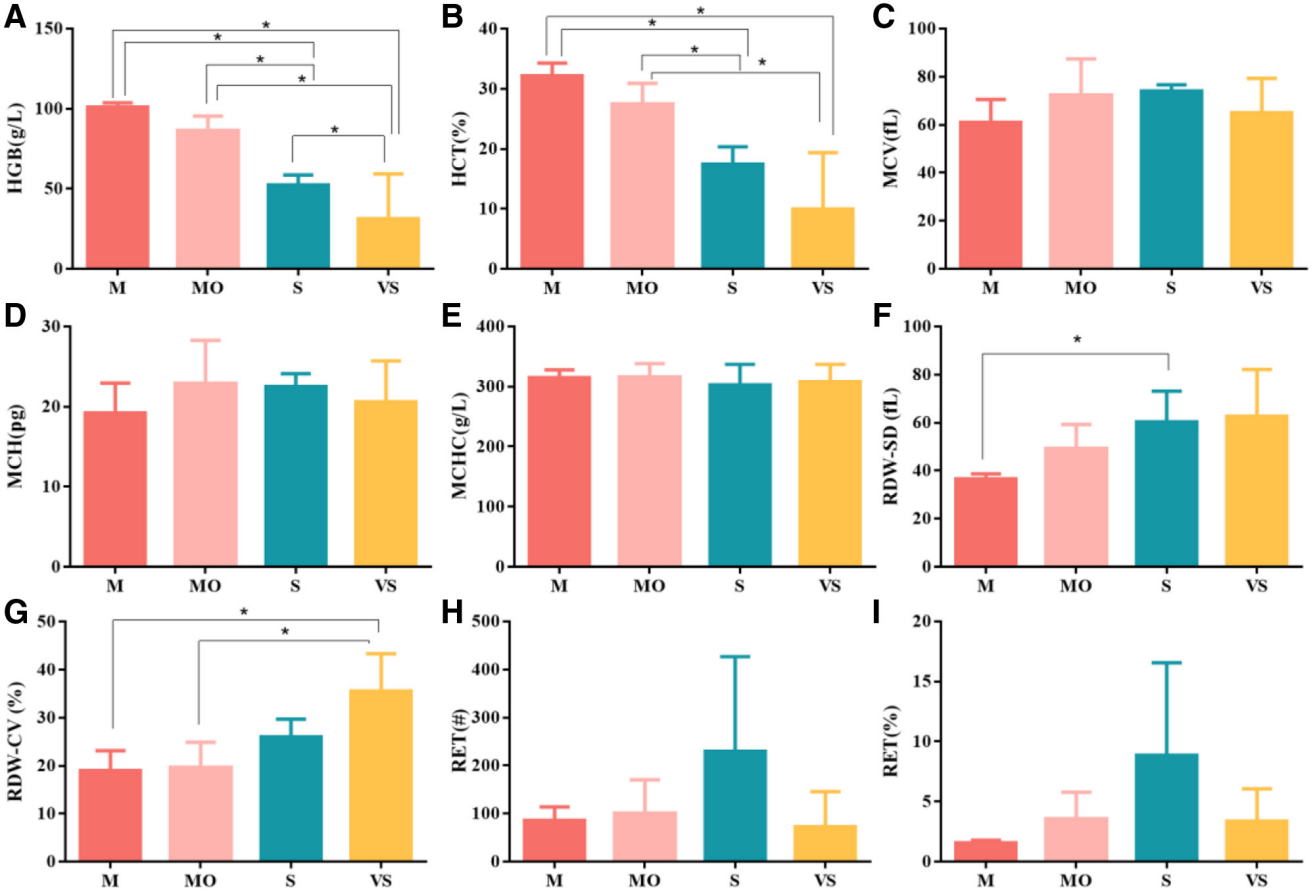
Hematological parameters across thalassemia severity groups. (A) HGB, (B) HCT, (C) MCV, (D) MCH, (E) MCHC, (F) RDW‐SD, (G) RDW‐CV, (H) RCT, and (I) RET. M: mild (HGB > 90 g/L); Mo: moderate (HGB 60–90 g/L); S: severe (HGB 30–60 g/L); VS: very severe (HGB < 30 g/L). Data are presented as mean ± SD. HGB levels showed a significant inverse correlation with disease severity (one-way ANOVA, *P* < .05). The very severe (VS) group exhibited greater variability in HGB. * denotes a significant difference (*P* < .05) between the indicated groups based on Tukey’s post hoc test. ANOVA = one-way analysis of variance, HGB = hemoglobin, HCT = hematocrit, MCH = mean corpuscular hemoglobin, MCHC = mean corpuscular hemoglobin concentration, MCV = mean corpuscular volume, RDW-CV = red cell distribution width coefficient of variation, RET = rearranged during transfection, SD = standard deviation.

Comparative analysis revealed no intergroup variations in erythrocyte indices (MCV, MCH, MCHC), or reticulocyte counts among the different severity classifications (all *P* > .05). However, red cell distribution width coefficient of variation, a measure of erythrocyte volumetric variation, was significantly elevated in the VS group compared with both the M and Mo groups (*P* < .05; Table 1, Fig. 1).

### 3.2. Genomic characteristics of INDEL variants in thalassemia

WES revealed a heterogeneous mutational spectrum across the 21 patients, with distinct patterns associated with disease severity. There were no significant differences in the INDEL/SNV burden or functional categories (frameshift, stopgain) across severity groups (all *P *> .05; Tables 2 and 3). Comparative analysis of INDEL variants across thalassemia severity groups revealed no statistically significant differences in coding sequence (CDS), frameshift, or non-frameshift mutations (all *P* > .05). Likewise, total INDEL burden (*P* > .05), zygosity distribution (homozygote, heterozygote), and variant annotation characteristics (Database of Single Nucleotide Polymorphisms percentage, novel variants) across severity groups showed no significant variation. While total INDEL counts were comparable (~5400–5500), a modest elevation in 3′ UTR variants was noted in the VS group (VS: 266.67 ± 8.96) relative to milder groups (M/Mo/S: 250–254). No severity-dependent trends were observed for functional variants (stopgain/stoploss) or noncoding regions (noncoding RNA, intronic). Results of INDEL detection and statistical analyses are shown in Table 2. Total INDEL counts remained remarkably consistent across severity groups (range: 5418.71–5502.67), with a non-statistically significant slight increase in the VS group (5502.67 ± 94.25). These findings indicate that INDELs contributed minimally to the observed heterogeneity in thalassemia severity within this cohort.

**Table 2:** Number of INDELs in different genomes and coding regions.

Type	M	Mo	S	VS
CDS	501.33 ± 2.89	493.29 ± 18.95	490.25 ± 8.41	490 ± 17.69
frameshift_deletion	85 ± 6.08	85.29 ± 10.03	84.75 ± 7.92	85.33 ± 5.03
frameshift_insertion	71.67 ± 1.53	71.57 ± 6.35	65.5 ± 5.13	67.33 ± 3.21
nonframeshift_deletion	120.33 ± 5.77	115.57 ± 4.79	117.13 ± 8.98	115.67 ± 6.51
nonframeshift_insertion	131.33 ± 1.15	127.29 ± 6.26	127.5 ± 6.3	126 ± 2.65
stopgain	6 ± 2.65	4.57 ± 0.98	5.75 ± 0.89	4 ± 2.65
stoploss	1 ± 0	0.86 ± 0.69	0.5 ± 0.53	0.33 ± 0.58
unknown	86 ± 2	88.14 ± 2.48	89.13 ± 2.95	91.33 ± 3.79
intronic	3772 ± 58.51	3754.71 ± 78.81	3801.63 ± 27.2	3833.67 ± 68.88
3′ UTR	253.67 ± 1.15	250 ± 6.61	250.63 ± 9.86	266.67 ± 8.96
5′ UTR	131.67 ± 4.93	129.43 ± 10.6	129.88 ± 9.52	124 ± 9.54
splicing	84.33 ± 3.51	80.57 ± 2.64	83.13 ± 2.59	81.33 ± 3.21
ncRNA_exonic	155.33 ± 7.57	156 ± 9.2	154.88 ± 4.61	153.33 ± 0.58
ncRNA_intronic	231 ± 12.29	242.29 ± 12.82	242.13 ± 14.44	243 ± 2.65
ncRNA_splicing	0.33 ± 0.58	0.43 ± 0.53	0.63 ± 0.52	0.33 ± 0.58
upstream	79.33 ± 4.73	76 ± 5.42	75.75 ± 3.45	79.33 ± 3.21
downstream	21 ± 5	22.57 ± 2.51	21.38 ± 2.92	20.33 ± 5.51
intergenic	210.67 ± 8.96	213.43 ± 12.23	209.38 ± 16.32	210.67 ± 7.64
Total	5440.67 ± 74.19	5418.71 ± 111.38	5459.63 ± 43.87	5502.67 ± 94.25

CDS = coding sequence, INDELs = insertions/deletions, M = mild, Mo = moderate, ncRNA = noncoding RNA, S = severe, UTR = untranslated region, VS = very severe.

**Table 3:** Characteristics of INDELs in the genome.

Type	M	Mo	S	VS
Total	5440.67 ± 74.19	5418.71 ± 111.38	5459.63 ± 47.02	5502.67 ± 94.25
Homozygote	1926 ± 16.52	1948.57 ± 32.33	1934 ± 50.13	1982.67 ± 45.83
Heterozygote	3514.67 ± 59.1	3470.14 ± 110.31	3525.63 ± 85.02	3520 ± 51.03
dbSNP_percentage (%)	94.12 ± 0.11	94.39 ± 0.33	94.33 ± 0.3	94.06 ± 0.07
Novel	320 ± 8.54	304.29 ± 23.91	309.5 ± 18.28	327 ± 2.65

dbSNP = Database of Single Nucleotide Polymorphisms, INDELs = insertions/deletions, M = mild, Mo = moderate, VS = very severe.

### 3.3. Genomic characteristics of SNVs across thalassemia

The SNV detection and statistical results are shown in Tables 4 and 5. CDS SNVs exhibited minimal variation across severity groups, ranging from 20,076.33 to 20,218. The ratio of synonymous to nonsynonymous variants remained consistent; synonymous variants constituted 51.1% to 51.2% of CDS SNVs (10,238.67–10,328), while nonsynonymous variants represented 46.1% to 46.2% (9259.33–9325). Stop-gain variants constituted 0.35% to 0.38% of CDS variants (70.75–77.33), and stop-loss variants were infrequent (7.57–9.00). Intronic variants constituted the most abundant category (21,545.67–21,757.67). Variants in UTRs showed minimal fluctuation: 3′ UTR variants numbered between 1414 and 1448, and 5′ UTR variants between 1030.33 and 1068. Noncoding RNA exonic variants also maintained stable counts (1385–1388.33).

**Table 4:** Number of single-nucleotide variants (SNVs) in different genomes and coding regions.

Type	M	Mo	S	VS
CDS	20,218 ± 213.72	20,099.14 ± 119.3	20,126.13 ± 170.09	20,076.33 ± 38
synonymous_SNV	10,328 ± 93.95	10,242.14 ± 35.93	10,256.88 ± 60.79	10,238.67 ± 62.15
nonsynonymous_SNV	9325 ± 121.39	9304.14 ± 94.34	9314.63 ± 125.39	9259.33 ± 81.64
stopgain	77.33 ± 2.31	72.57 ± 4.72	70.75 ± 5.18	74 ± 2.65
stoploss	9 ± 1	7.57 ± 1.81	8.25 ± 2.12	7.67 ± 1.53
unknown	478.67 ± 40.53	472.71 ± 31.62	475.63 ± 36.92	496.67 ± 22.28
intronic	21,757.67 ± 218.29	21,674.86 ± 118.81	21,639 ± 147.29	21,545.67 ± 108.9
3′ UTR	1448 ± 23.9	1414 ± 38.96	1418.13 ± 15.7	1423 ± 28.69
5′ UTR	1068 ± 22.27	1032.43 ± 25.36	1040.5 ± 24.11	1030.33 ± 18.72
splicing	54.67 ± 4.04	53.57 ± 5.5	54 ± 3.46	49.67 ± 1.53
ncRNA_exonic	1388.33 ± 4.73	1380.86 ± 26.11	1387.5 ± 32.87	1385 ± 32.08
ncRNA_intronic	1319.33 ± 8.5	1334.57 ± 35.99	1315.13 ± 17.64	1298.33 ± 31.97
ncRNA_splicing	4.33 ± 0.58	4 ± 1.29	3.88 ± 2.42	3.33 ± 0.58
upstream	504 ± 23.81	497.29 ± 19.01	494.88 ± 22.4	477 ± 15.1
downstream	162 ± 6.56	160 ± 11.31	167.75 ± 13.59	158 ± 6.08
intergenic	1795 ± 56.03	1830 ± 56.83	1807.13 ± 47.2	1805.33 ± 41.48
Total	49,719.33 ± 451.6	49,480.71 ± 311.34	49,454 ± 287.83	49,252 ± 192.29

CDS = coding sequence, M = mild, Mo = moderate, ncRNA = noncoding RNA, S = severe, UTR = untranslated region, VS = very severe.

**Table 5:** Characterization of SNVs in the genome.

Type	M	Mo	S	VS
Total	49,719.33 ± 451.6	49,480.71 ± 311.34	49,454 ± 287.83	49,252 ± 192.29
Homozygote	20,322 ± 24.27	20,444.86 ± 372.95	20,386.88 ± 332.27	20,426.33 ± 427.39
Heterozygote	29,397.33 ± 475.87	29,035.86 ± 502.58	29,067.13 ± 467.45	28,825.67 ± 242.29
dbSNP_percentage	99.45 ± 0.02	99.44 ± 0.04	99.44 ± 0.04	99.45 ± 0.04
Ts	35,496.33 ± 315	35,255.57 ± 208.6	35,252.5 ± 184.51	35,120.33 ± 86.12
Tv	14,223 ± 149.61	14,225.14 ± 132.35	14,201.5 ± 113.29	14,131.67 ± 120.21
Ts/Tv	2.49 ± 0.02	2.48 ± 0.02	2.48 ± 0.01	2.48 ± 0.02
Novel	274.33 ± 6.81	277.57 ± 19.48	277.75 ± 21.89	271.67 ± 22.81
Novel_Ts	166.67 ± 9.71	162.14 ± 14.45	163.25 ± 10.28	161.33 ± 14.01
Novel_Tv	107.67 ± 9.24	115.43 ± 8	114.5 ± 14.19	110.33 ± 10.07
Novel_Ts/Tv	1.56 ± 0.22	1.41 ± 0.12	1.44 ± 0.16	1.46 ± 0.09

dbSNP = Database of Single Nucleotide Polymorphisms, M = mild, Mo = moderate, S = severe, SNVs = single-nucleotide variants, Ts/Tv = transition/transversion, VS = very severe.

**Figure 2.**
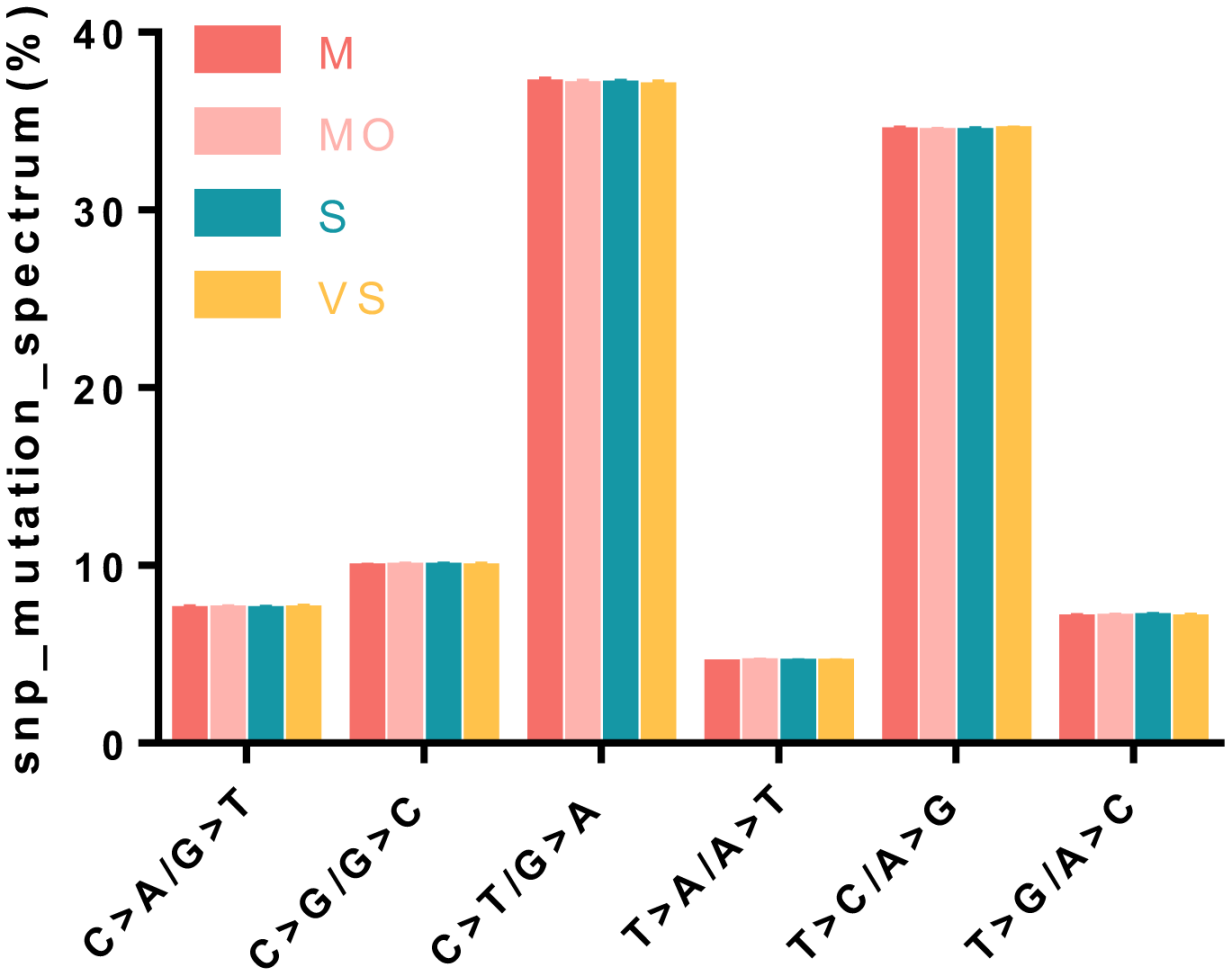
Distribution of single-nucleotide polymorphism (SNP) mutation types. The mutation spectrum is shown as the percentage of each substitution type among all SNVs. C>T/G>A and T>C/A>G were the most frequent transitions across all severity groups. No significant differences in the mutation spectrum were observed among the 4 groups (*P* > .05), indicating that disease severity grouping did not affect the overall distribution of SNP types. M = mild, Mo = moderate, S = severe, SNVs = single-nucleotide variants, VS = very severe.

A modest reduction in total SNVs was observed in the VS group (49,252 ± 192.29) compared with the M group (49,719.33 ± 451.60; *P* < .05). C>T/G>A and T>C/A>G were the most frequent SNP mutation types across all 4 groups. No significant differences in the SNP mutation spectrum were observed among the 4 groups, indicating that disease severity grouping did not affect the overall distribution of SNP types (Fig. 2). Heterozygous SNVs were predominant (58.1%–59.1%). Annotation against the Database of Single Nucleotide Polymorphisms exceeded 99.4% in all groups. The global transition/transversion ratios were also consistently high, ranging from 2.48 to 2.49. Novel variants comprised 0.55% to 0.56% of the total SNVs (271.67–277.75) and displayed a lower transition/transversion ratio (1.41–1.56) than the overall genomic ratio.

In a targeted analysis prompted by the observation of potential regulatory effects, we examined the relationship between 3′ UTR SNVs and MCHC. Patients carrying any 3′ UTR variant (n = 14) had a mean MCHC of 301 ± 25 g/L, whereas those without (n = 7) had a mean MCHC of 321 ± 18 g/L. This difference was statistically significant (unpaired *t* test, *P* = .008), supporting a potential role for these variants in modulating erythrocyte HGB concentration.

### 3.4. Mutation spectrum and genotype–phenotype correlations identified by WES

The gene mutation profiles of the 21 cases analyzed by WES are summarized in Table 6. Classical *HBB* mutations were identified in 18 cases (85.7%).

**Table 6:** Mutation types in patients with thalassemia from Huadu identified by WES.

No.	Gene of mutation	Results
FXY	*HBB*	CD41/42(-TTCT) β^0^
LZR	*HBB*	Codon 17(A>T) β^0^
YJW	*HBB*	Initiation codon ATG>AGG β^0^
ZHS	*HBB*	c.-28A>Gβ^+^
ZZF*	*HBB*/*MYB*	CD41/42(-TTCT) β^0^, IVS-II-654(C>T) β^+^/NM_001130173.2:c.107G>A(p.R36H)
BWS	*HBB*	CD41/42(-TTCT) β^0^, IVS-II-654(C>T) β^+^
QZY	*HBB*	CD41/42(-TTCT) β^0^
WKT	*HBB*	CD41/42(-TTCT) β^0^
YGX*	*HBB*/*HBD*	IVS-II-654(C>T) β^+^/NM_000519.4:c.440A>T(p.H147L)
PCH*	HBG1	NM_000559.3:c.364G>T:p.E122*
CXM	*HBA2*	Hb Constant Spring (CS)α+
DDN	*HBB*	CD 108(A>C) Hb Shizuoka
FDQ	*HBA2*	Hb Constant Spring (CS)α+
ZWZ	*HBB*	Codons 41/42(-TTCT) β^0^, Codon 26(G>A) Hb E
SY	*HBB*	Codons 41/42(-TTCT) β^0^, c.-28A>Gβ^+^
LRX	*HBB*	IVS-II-654(C>T) β^+^
CWY	*HBB*	CD41/42(-TTCT) β^0^, CD27/28(+C) β^0^
HWL	*HBA2*	Hb Constant Spring (CS) α+
HDC	*HBB*	CD41/42(-TTCT) β^0^, c.-28A>Gβ^+^
LJC	*HBB*	IVS-II-654(C>T) β^+^, c.-28A>Gβ^+^
CHH	*HBB*	c.-28A>Gβ^+^

* Rare occurrence.

In these cases, the principal added value of WES was the ability to perform comprehensive genotyping in a single assay and to identify co-inherited genetic modifiers. The inclusion of these patients in our consecutive-case cohort was based on their clinical presentation of unexplained anemia or a relevant family history, demonstrating that WES can provide confirmatory and additional genetic information even when a primary mutation is detectable by standard methods.

Additionally, 3 rare non-β-globin variants were detected:

Case ZZF: This patient carried heterozygous *HBB* CD41/42(-TTCT) β^0^ and IVS-II-654(C>T) β^+^ mutations. A *MYB* variant, NM_001130173.2:c.107G>A (p.R36H), was also detected. This variant is absent from the Genome Aggregation Database, and its significance is uncertain, though it may be a potential modulator of fetal HGB (HbF) expression.
Case YGX: This patient was heterozygous for *HBB* IVS-II-654(C>T) β^+^ and carried a known δ-thalassemia variant, *HBD* c.440A>T (p.H147L; HbA_2_-Yialousa). This variant is known to suppress HbA_2_ expression and may lead to underdiagnosis of β-thalassemia.
Case PCH: The patient carried a heterozygous nonsense mutation in *HBG1*, NM_000559.3:c.364G>T (p.E122*; combined annotation-dependent depletion = 38), predicted to truncate the γ-globin chain. The phenotype was further complicated by concurrent iron deficiency and α-thalassemia.

Several cases exhibited genotype–phenotype discrepancies: Case CHH (VS) was classified as VS but carried only a heterozygous *HBB* c.-78A>G (β^+^) mutation. The severity may reflect an undetected second variant (e.g., a structural variant not captured by WES), a co-inherited genetic modifier, or an acquired comorbidity. Cases DDN and LRX presented with severe anemia (HGB < 60 g/L) but carried only 1 *HBB* mutation; concurrent iron deficiency likely exacerbated their anemia, highlighting the importance of incorporating iron status into clinical assessment. Cases CXM, FDQ, and HWL, all carriers of the Hb Constant Spring variant, showed more severe anemia than typically expected, suggesting a possible co-inherited α-globin gene lesion not detectable by WES.

These detailed findings illustrate the potential of WES to uncover genetic complexity beyond primary hemoglobinopathies, offering insights into potential disease modifiers and diagnostic pitfalls.

## 4. Discussion

This study used WES to investigate the genetic landscape of thalassemia in the Huadu population, revealing classical *HBB* mutations and rare variants in modifier genes that contribute to phenotypic heterogeneity. Our key finding is that although WES provided a comprehensive genetic diagnosis or valuable insights in all patients, its principal added value over conventional methods lies in the detection of rare non-*HBB* variants and its capacity to resolve complex genotypes within a single test. However, our data do not support the use of WES as a universal first-line screening tool.

A significant inverse correlation was observed between disease severity and HGB levels (*P* < .05), consistent with established clinical paradigms. Notably, the VS group exhibited greater variability in HGB levels, reflecting the complex clinical heterogeneity observed in advanced thalassemia phenotypes.

Genomic analyses revealed a largely uniform burden of INDELs and SNVs across all severity groups, with no statistically significant differences in coding or functional variant categories. This suggests that overall mutational burden does not fully explain the observed variability in clinical severity. However, our targeted analysis identified a significant association between the presence of 3′ UTR SNVs and lower MCHC levels. This finding highlights the potential contribution of regulatory-region alterations to the hematological phenotype and warrants further functional investigation.

Importantly, WES identified rare mutations in 3 modifier genes, that is, *MYB*, *HBD*, and *HBG1*, in cases ZZF, YGX, and PCH, respectively, each of which may influence disease expression independently of classical β-globin mutations.

The *MYB* gene mutation NM_001130173.2:c.107G>A (p.R36H), identified in case ZZF, is a variant of uncertain significance. *MYB* is a key transcriptional regulator involved in HbF suppression, and variants within the *HBS1L*–*MYB* intergenic region are known to modulate HbF levels, potentially ameliorating the severity of β-thalassemia.^[7,8]^ While the p.R36H substitution is not currently actionable and routine screening is not recommended, its detection highlights the potential for future pharmacogenetic insights (e.g., thalidomide responsiveness) as functional evidence accumulates.

The *HBD* mutation NM_000519.4:c.440A>T (p.H147L) in case YGX is a known delta-thalassemia variant that suppresses HbA_2_ production.^[9]^ This reduction can result in falsely normal HbA_2_ levels, leading to misdiagnosis of β-thalassemia carriers – a well-documented pitfall in hemoglobinopathy diagnostics. As demonstrated by Zakaria et al, such cases require genetic testing to avoid false-negative results and potential misdiagnosis of β-thalassemia carriers.^[9]^ This case underscores the necessity of genetic testing when there is a high index of suspicion despite normal HbA_2_ levels.

Case PCH carried a heterozygous *HBG1* mutation (NM_000559.3:c.364G>T, p.E122*), which primarily affects HbF.^[10]^ The patient’s phenotype was further complicated by concurrent iron deficiency and α-thalassemia. Literature suggests that *HBG1* mutations have a minimal independent impact on the clinical severity of α-thalassemia but may influence HbF expression. This case highlights the importance of a comprehensive assessment, including iron studies, to accurately interpret the contribution of each genetic and acquired factor to the overall anemia.

Collectively, these findings highlight the complex genetic architecture underlying thalassemia severity. Rare variants in modifier genes such as *MYB*, *HBD*, and *HBG1* contribute to nuanced effects that may alter clinical presentation, diagnostic accuracy, and potentially, therapeutic responsiveness. The implementation of WES in routine diagnostics facilitates the detection of such variants, supporting precision medicine approaches tailored to individual genetic profiles.^[11]^

To incorporate these findings into clinical practice, we propose a refined, 2-tiered screening strategy. In the first tier, cost-effective conventional methods (hematological indices, HGB electrophoresis, gap polymerase chain reaction) would remain the frontline tools for detecting the majority of common thalassemia carriers. Patients with suggestive hematological features but negative or equivocal conventional results, as well as individuals from high-risk families with a history of severe thalassemia of unknown genotype, would then progress to the second tier: comprehensive genetic testing using WES or targeted next-generation sequencing panels. These advanced assays should be designed to interrogate not only the coding regions of globin genes but also essential regulatory elements and modifier genes.

This study has several limitations. First, the relatively small sample size (n = 21) from a single district may limit the generalizability of our findings. The rare variants identified (*MYB*, *HBD*, *HBG1*) are likely specific to this local population. Second, the lack of orthogonal validation (e.g., Sanger sequencing) for WES-detected variants is a technical limitation, although mitigated by rigorous bioinformatic quality control and Integrated Genomics Viewer review. Third, the functional impacts of prioritized variants (e.g., *MYB* p.R36H) remain unconfirmed. Finally, we did not perform a formal cost–benefit analysis, which is essential for assessing the feasibility of implementing WES in resource-limited settings where conventional screening remains substantially more cost-effective. Future multicenter studies with larger cohorts, orthogonal validation, functional assays, and health-economic evaluations are needed to validate and extend these findings.

## 5. Conclusion

This study demonstrates the utility of WES in identifying both classical and rare modifier gene mutations that contribute to the clinical heterogeneity of thalassemia in the Huadu population. Our findings highlight a significant inverse relationship between HGB levels and disease severity. Notably, we identified a significant association between 3′ UTR SNVs and reduced MCHC, as well as rare variants in *MYB*, *HBD*, and *HBG1* as potential modulators of disease expression, with implications for diagnosis and genetic counseling. These results support the selective integration of WES into standard thalassemia diagnostic workflows to enhance precision diagnosis and inform individualized management strategies. Future research should prioritize functional characterization of noncoding variants and modifier genes to further elucidate genotype–phenotype correlations and refine therapeutic interventions.

## Acknowledgments

The authors wish to express their gratitude for the facility support provided by Guangzhou Huadu District People’s Hospital, China. We also thank the patients and their families for their participation in this study. All individuals named in this section have provided their permission to be named.

## Author contributions

**Conceptualization:** Run Guowei, Jiang Yan, Huang Yulan, Ji Linhua.

**Data curation:** Run Guowei, Jiang Yan, Jiang Changlv, Yu Bizhen, Bi Jingnan, Tan Cuijin, Lei HaoHao.

**Formal analysis:** Run Guowei, Jiang Yan, Jiang Changlv, Yu Bizhen, Bi Jingnan, Tan Cuijin, Lei HaoHao, Ji Linhua.

**Methodology:** Run Guowei, Xu Jingxia, Zeng Lihua, Ji Linhua.

**Supervision:** Ji Linhua.

**Writing – original draft:** Run Guowei, Jiang Yan.

**Writing – review & editing:** Ji Linhua.
